# CD8^+^ T Lymphocyte Coexpression Genes Correlate with Immune Microenvironment and Overall Survival in Breast Cancer

**DOI:** 10.1155/2021/5533923

**Published:** 2021-03-27

**Authors:** Jialing Jiang, Yi Zhao

**Affiliations:** Department of Oncology, Shengjing Hospital of China Medical University, Shenyang 110004, China

## Abstract

**Purpose:**

To identify CD8+ T lymphocyte-related coexpressed genes that increase CD8+ T lymphocyte proportions in breast cancer and to elucidate the underlying mechanisms among relevant genes in the tumor microenvironment.

**Method:**

We obtained breast cancer expression matrix data and patient phenotype following information from TCGA–BRCA FPKM. Tumor purity, immune score, stromal score, and estimate score were calculated using the estimate package in R. The CD8^+^ T lymphocyte proportions in each breast carcinoma sample were estimated using the CIBERSORT algorithm. The samples with *p* < 0.05 were considered to be significant and were taken into the weighted gene coexpression network analysis. Based on the CD8^+^ T lymphocyte proportion and tumor purity, we generated CD8^+^ T lymphocyte coexpression networks and selected the most CD8^+^ T lymphocyte-related module as our interested coexpression modules. We constructed a CD8+ T cell model based on the least absolute shrinkage and selection operator method (LASSO) regression model and robust model and evaluate the prediction ability in different subgroups.

**Results:**

A breast carcinoma CD8+ T lymphocyte proportion coexpression yellow module was determined. The coexpression genes in the yellow module were determined to increase the CD8+ T lymphocyte proportion levels in breast cancer patients. The yellow module was significantly enriched in the antigen presentation process, cellular response to interferon-gamma, and leukocyte proliferation. Subsequently, we generated CD8+ T cell-related genes lasso regression risk model and robust model, and eight genes were taken into the risk model. The risk score showed significant prognostic ability in various subgroups. Expression levels of proteins, encoded by CD74, were lower in the breast carcinoma samples than in normal tissue, suggesting expression differences at both the mRNA and the protein levels.

**Conclusion:**

These eight CD8+ T lymphocyte proportion coexpression genes increase CD8^+^ T lymphocyte in breast cancer by an antigen presentation process. The mechanism might suggest new pathways to improve outcomes in patients who do not benefit from immune therapy.

## 1. Introduction

Breast cancer has entered the era of individualized treatment by way of molecular classification. In addition to traditional surgery, chemotherapy, and radiotherapy, breast cancer treatment includes endocrine therapy, molecular targeted therapy, and immunotherapy [1]. Of these, immunotherapy has achieved remarkable effects in the treatment of a variety of malignant tumors. However, immunotherapy for breast cancer is a recent development. Breast cancer is a “cold” tumor in terms of immunotherapy, the exploratory studies of PD-1/PD-L1 inhibitors using monotherapy were absent, and the population that benefits is very limited [1]. At present, the most critical issue in breast cancer immunotherapy is the issue of selection of the appropriate population and reasonable treatment mode, so that more patients can benefit from immunotherapy, survival can be extended, and quality of life can be improved.

Lack of CD8+ tumor-infiltrating lymphocytes, low PD-1 expression, and tumor mutation burden factors are thought to be the primary influencing factors leading to insensitivity to immunotherapy in advanced breast cancer. In triple-negative breast cancer, the positive expression rate of PD-L1 is about 20%, which is higher than that of other subtypes of breast cancer [2]. An advanced breast cancer analysis suggested that PD-L1 is not only related to advanced breast cancer prognosis, and it is also a biomarker for screening suitable populations for immunotherapy [3]. A meta-analysis involving 8583 breast cancer patients of various subtypes suggested that PD-L1 overexpression is significantly negatively correlated with the overall survival of patients, and the mechanism may be that high PD-L1 expression promotes breast cancer immune escape [4]. Although clinical immunohistochemistry can be used to assess tumor PD-L1 expression levels, there remain limitations to using PD-L1 expression as a biomarker of immunotherapy sensitivity. Tumor PD-L1 expression is heterogeneous [5] and is affected by previous treatments such as chemotherapy and radiotherapy [6].

Tumor-infiltrating lymphocytes are polymorphic, mainly existing in the microenvironment of tumor tissues; they include CD4+, CD8+ T cells, B cells, and NK cells [7]. Studies have shown that there are more TILs in the tumor microenvironment of HER2-positive breast cancer and this is related to prognosis [8]. In a meta-analysis of non-small cell lung cancer, more CD8+ TILs were associated with improvement in overall survival [9]. In patients with advanced melanoma treated with pembrolizumab, the density of CD8+ T cells in the invasion margin and tumor center of the tissue specimens of responders was higher than those of nonresponders [10].

In the present study, we hypothesized that increasing the content of CD8+ T lymphocytes would improve outcomes after immunotherapy. By constructing a coexpression network of CD8+ T lymphocyte content, we explored the biological functions and related coexpression factors that are most related to CD8+ lymphocyte content.

## 2. Method

### 2.1. CD8+ T Cell Proportion, Tumor Purity, and Tumor Mutation Burden

We downloaded The Cancer Genome Atlas TCGA–BRCA FPKM data (http://cancergenome.nih.gov/) containing 1097 samples (Supplementary Table 1). GSE78220 [11] was also download from the GEO database. We evaluated CD8+ T lymphocyte cell proportions based on the LM22 matrix using the CIBERSORT [12] algorithm. Breast tissue samples with *p* < 0.05 were considered to be significant and were taken into the subsequent analysis. The Estimation of Stromal and Immune cells in Malignant Tumor tissues using Expression data (ESTIMATE) is a method that infers the fraction of stromal and immune cells using gene expression signatures [13]. Using the ESTIMATE package, we calculated tumor purity in each breast cancer sample. Tumor mutation burden (TMB) per megabyte was calculated by dividing the total number of mutations by the size of the target coding region [14, 15].

### 2.2. Coexpression Network Generation

Weighted gene coexpression network analysis (WGCNA) is a system biology method that transforms correlations into connection weights or topology overlap values [16]. We used this method to generate a CD8+ T lymphocyte proportion coexpressing network. The expression patterns are similar for genes with the same pathway and biological effect [17, 18]. In this study, we built a scale-free topology network; set the soft threshold at 5, *R* square = 0.93, slope = −2.09; we set the number of genes in the minimum module at 30. The CD8+ T lymphocyte proportion was considered for phenotype files in the WGCNA analysis. In this manner, a cluster of CD8+ T lymphocyte proportion-related genes with a similar pathway process was determined in the same module. We identified factors with CD8+ T lymphocyte correlation greater than 0.4 and module correlation greater than 0.6 in the most coexpression modules.

### 2.3. Function Enrichment and Protein-Protein Network of Coexpression Module

The Database for Annotation, Visualization and Integrated Discovery (DAVID, v6.8) is an open-source database that performs function enrichment [19]. The Kyoto Encyclopedia of Genes and Genomes (KEGG) [20] (https://www.genome.jp/kegg/) and Gene Ontology (GO) [21] (http://geneontology.org/) analysis were applied to determine the biological function, cellular component, and molecular function in each coexpression module. Cytoscape was used to conduct the protein-protein interaction network for the coexpression genes.

### 2.4. CD8+ T Lymphocyte Genes Prognostic Value

The Kaplan–Meier analysis was used to calculate the clinical outcome significance of these CD8+ T lymphocyte coexpression genes. Subsequently, the least absolute shrinkage operator (LASSO) and robust prognosis model was applied to conduct CD8+ T lymphocyte coexpression genes prognostic model. We evaluated the CD8+ T lymphocyte coexpression genes prognosis model in various subgroups. Finally, we calculated the difference of these coexpression genes in various subgroups, including tumor purity, survival status, and TMB.

### 2.5. Gene Set Enrichment Analysis and the Human Protein Atlas Database

Gene Set Enrichment Analysis (GSEA) [22] was used to calculate the most involved pathway to these coexpression genes. The Human Protein Atlas (HPA) (http://www.proteinatlas.org) [23] was used to demonstrate differences in coexpressing genes at the protein level.

## 3. Results

**Figure 1:**
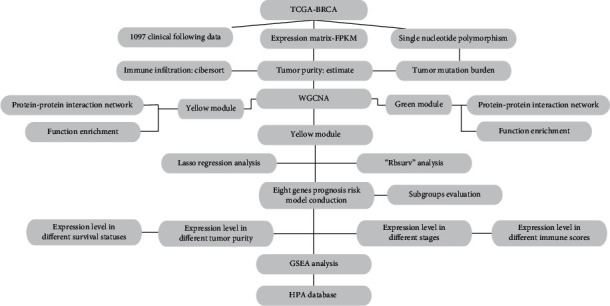
The flow chart of the experimental sequence.

The flow chart of the experimental protocol is shown in Figure 1.

### 3.1. CD8+ T Lymphocyte, Tumor Purity, and Tumor Mutation Burden Evaluation

**Figure 2:**
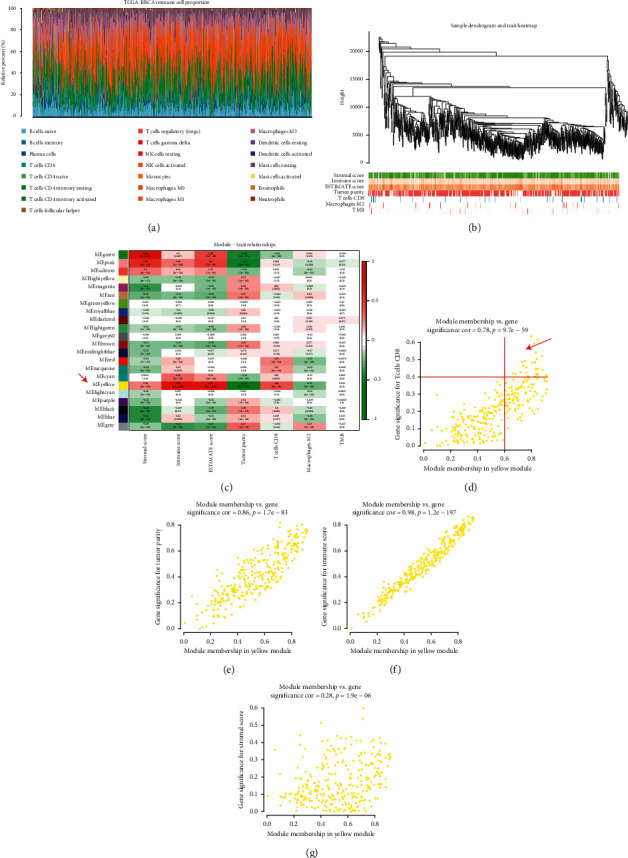
(a) We evaluated 860 breast cancer samples accurately using CD8+ T lymphocytes. (b) Hierarchical clustering tree was built using the dynamic hybrid cutting method. (c) Twenty-two coexpression models were identified. The yellow module had the strongest correlation with CD8+ T lymphocyte proportion in the TCGA-BRCA cohort (Cor = -0.41; *p* = 1*e* − 28). (d) The yellow module showed a significant correlation to CD8+ T Cell (Cor = 0.78, *p* = 9.7*e* − 259), (e) tumor purity (Cor = 0.86, *p* = 1.7*e* − 83), (f) immune score (Cor = 0.98, *p* = 1.2*e* − 197), and (g) stomal score (Cor = 0.28, *p* = 1.9*e* − 06).

We obtained the tumor purity, matrix score, immune score, and tumor mutation burden corresponding to each sample. Using the screening principle of *p* < 0.05, we obtained 860 breast cancer samples accurately evaluated by CD8+ T lymphocytes (Figure 2(a)). By integrating the immune microenvironment scoring file with the CD8+ T lymphocyte content samples, we determined WGCNA's phenotype entry files.

### 3.2. CD8+ T Lymphocyte Coexpression Network Conduction in TCGA

Weighted gene coexpression network analysis (WGCNA) was performed using TCGA–BRCA. A hierarchical clustering tree was built using the dynamic hybrid cutting method (Figure 2(b)); 22 coexpression models were identified (Figure 2(c)). The correlation coefficients among CD8+ T lymphocyte proportion, tumor purity, TMB, and coexpression modules are shown in Figure 2(c). The yellow module had the strongest correlation with CD8+ T lymphocyte proportion in the TCGA - BRCA cohort (Cor = -0.41; *p* = 1*e* − 28) (Figure 2(c)). Based on these findings, we supplemented the heat map of the correlation between the factors in the yellow module (Figures 2(d)–2(g)). The yellow module showed a significant correlation with CD8+ T cell (Cor = 0.78, *p* = 9.7*e* − 59), tumor purity (Cor = 0.86, *p* = 1.7*e* − 83), immune score (Cor = 0.98, *p* = 1.2*e* − 197), and stomal score (Cor = 0.28, *p* = 1.9*e* − 06).

### 3.3. CD8+ T Lymphocyte Coexpression Module Functional Enrichment

**Figure 3:**
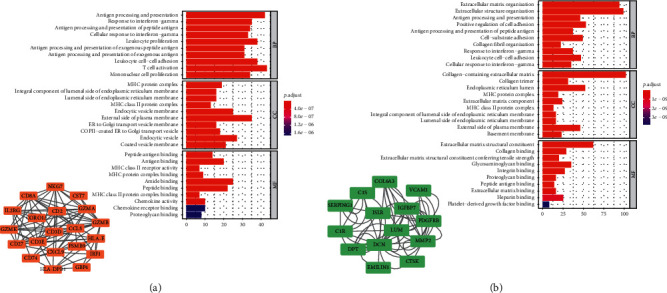
(a) The 28 CD8+ T lymphocyte proportions positively coexpressing mRNA were most significantly enriched in the antigen processing and presentation and response to interferon-gamma, which suggested these biological processes might promote CD8+ T lymphocyte infiltration in the breast cancer microenvironment. (b) The CD8+ T lymphocyte negatively coexpressing module was most significantly enriched in the extracellular matrix organization.

We determined 28 CD8+ T lymphocyte proportions positively coexpressing mRNA with coefficient > 0.4 in the TCGA-BRCA yellow module (Table 1). The 28 CD8+ T lymphocyte proportions positively coexpressing mRNA were most significantly enriched in the antigen processing and presentation and response to interferon-gamma, suggesting that these biological processes might promote CD8+ T lymphocyte infiltration in the breast cancer microenvironment (Figure 3(a)). The CD8+ T lymphocyte negatively coexpressing module was most significantly enriched in the extracellular matrix organization (Figure 3(b)). The protein-protein interaction network of the yellow module and green module is shown in Figure 3.

### 3.4. Clinical Outcome of CD8+ T Lymphocyte Infiltration-Related Genes

**Figure 4:**
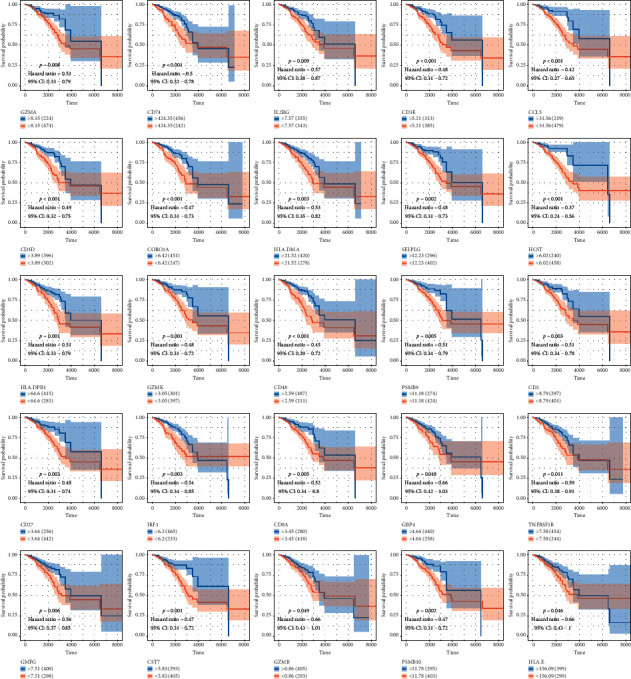
The patients in low expression groups for GZMA (TCGA :  *p* < 0.001), CD74 (TCGA : *p* < 0.001), IL2RG (TCGA : *p*=0.009), CD3E (TCGA : *p* < 0.001), CCL5 (TCGA : *p* < 0.001), CD3D (TCGA : *p* < 0.001), CORO1A (TCGA : *p* < 0.001), HLA-DMA (TCGA : *p*=0.003), SELPLG (TCGA : *p*=0.002), HCST (TCGA : *p* < 0.001), HLA-DPB (TCGA : *p*=0.001), GZMK (TCGA : *p*=0.001), CD48 (TCGA : *p* < 0.001), PAMB9 (TCGA : *p*=0.005), CD2 (TCGA : *p*=0.003), CD27 (TCGA : *p*=0.003), IRF1 (TCGA : *p*=0.003), CD8A (TCGA : *p*=0.005), GBP4 (TCGA : *p*=0.048), TNFRSF1B (TCGA : *p*=0.011), GMFG (TCGA : *p*=0.006), CST7 (TCGA : *p*=0.001), GZMB (TCGA : *p*=0.049), PSMB10 (TCGA : *p*=0.002), and HLA-E (TCGA : *p*=0.046) showed survival risk against high expression groups.

To demonstrate their significance on clinical outcomes, we performed a survival analysis. The patients in low expression groups for GZMA (TCGA :  *p* < 0.001), CD74 (TCGA : *p* < 0.001), IL2RG (TCGA : *p*=0.009), CD3E (TCGA : *p* < 0.001), CCL5 (TCGA : *p* < 0.001), CD3D (TCGA : *p* < 0.001), CORO1A (TCGA : *p* < 0.001), HLA-DMA (TCGA : *p*=0.003), SELPLG (TCGA : *p*=0.002), HCST (TCGA : *p* < 0.001), HLA-DPB (TCGA : *p*=0.001), GZMK (TCGA : *p*=0.001), CD48 (TCGA : *p* < 0.001), PAMB9 (TCGA : *p*=0.005), CD2 (TCGA : *p*=0.003), CD27 (TCGA : *p*=0.003), IRF1 (TCGA : *p*=0.003), CD8A (TCGA : *p*=0.005), GBP4 (TCGA : *p*=0.048), TNFRSF1B (TCGA : *p*=0.011), GMFG (TCGA : *p*=0.006), CST7 (TCGA : *p*=0.001), GZMB (TCGA : *p*=0.049), PSMB10 (TCGA : *p*=0.002), and HLA-E (TCGA : *p*=0.046) showed survival risk against high expression groups (Figure 4). These results suggest that these CD8+ T lymphocyte infiltration-related genes act in protective roles in breast cancer.

### 3.5. Lasso Regression Risk Model of CD8+ T Lymphocyte Coexpression Genes

**Figure 5:**
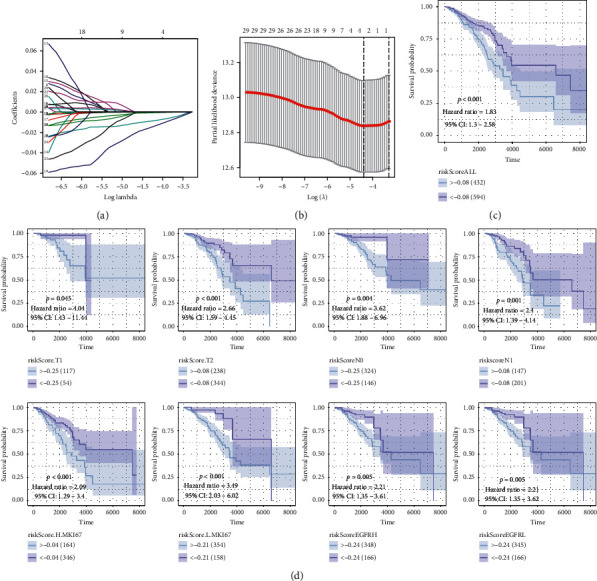
(a-b) Establishment of a risk signature using the lasso regression curve and verification. (c) The samples in high-risk score level samples showed worse clinical survival outcomes for breast cancer patients (TCGA : *p* < 0.001; HR = 1.83). (d) The risk score was evaluated in various subgroups, including gender, stage, metastasis status, Ki-67, and EGFR. The results were the of the same significance in these subgroups.

A CD8+ T lymphocyte coexpression gene lasso regression hazard model was conducted based on these breast cancer prognosis protective factors. Risk = –0.0017^*∗*^CD74–0.0128^*∗*^IRF1–0.0024^*∗*^CCL5+0.0055^*∗*^GIMAP4–0.027^*∗*^HCST–0.0064^*∗*^CST7–0.0037^*∗*^PSMB10–0.0002^*∗*^HLA–DMA. The samples in high-risk score level samples showed worse clinical survival outcomes for breast cancer patients (TCGA :*p* < 0.001; HR = 1.83) (Figure 5). The risk score was evaluated in various subgroups, including age, gender, stage, tumor purity, tumor mutation burden, metastasis status, Ki-67, and EGFR. The results were significant in these subgroups.

### 3.6. Robust Survival Analysis

Based on the 25 prognostic genes obtained from the coexpression network, we performed a robust survival analysis of these genes. To obtain the most stable prognostic model with the lowest degree of freedom, the Rbsurv package and AICs are used to select the prognostic model with the parameter as follows: iteration times = 100 and max concern genes = 20 (Table 2). ^*∗*^ in Table 2 represents prognostic genes included in the robust model. Later, we identified eight prognostic factors based on the common prognostic genes of the robustness model and the lasso model.

### 3.7. Clinical Phenotype and Immunophenotype

**Figure 6:**
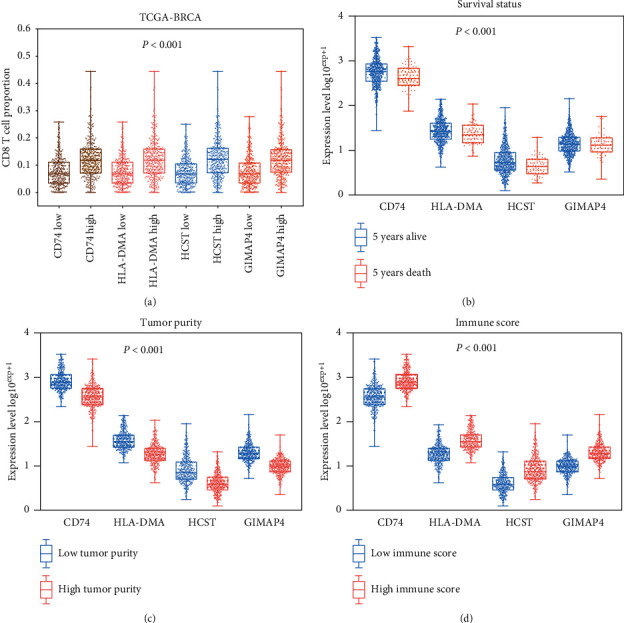
(a) The CD8+ T cell proportion level in different gene expression patterns. (b) The gene expression level in different survival statuses. (c) The gene expression level in different tumor purity. (d) The gene expression level in different immune scores.

**Figure 7:**
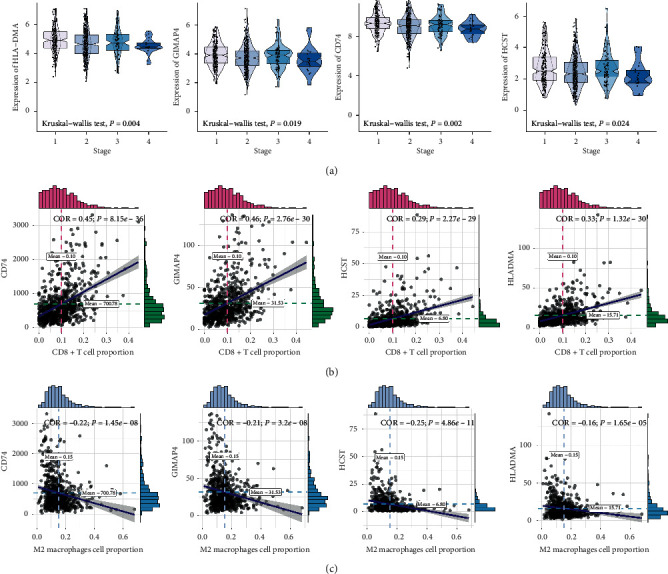
The correlation between CD8+ T cell coexpression genes and clinical stages (a), CD8+ T lymphocytes (b), and M2 macrophages (c).

Having defined a clinical prognostic risk propensity weighted score consisting of four factors, we then found that these factors were coexpressed with one another and were closely related to the level of CD8+ T lymphocyte infiltration. These factors affect outcomes. Then, to demonstrate the relationship between these factors and clinical phenotype and immunophenotype more specifically, we drew multiple sets of box plots. The content of CD8+ T lymphocytes in the high expression group of these four factors showed a higher level of infiltration, suggesting that our four factors and related biological processes promoted the infiltration of CD8+ T lymphocytes in tumor tissues (Figure 6(a)). The expression levels of genes in the 5-year mortality group were lower than those of the 5-year survival group, suggesting their protective effect on outcomes. This trend was the same as that of CD8+ T lymphocytes (Figure 6(b)). Then, we found that expression levels of these factors were low in the high tumor purity group, and these factors in the high immune score group were low (Figures 6(c) and 6(d)). These directly or indirectly indicate that these four factors promote the CD8+ T lymphocyte infiltration. We also drew a scatter plot of correlations with clinical stages (Figure 7(a)), CD8+ T lymphocytes (Figure 7(b)), and M2 macrophages (Figure 7(c)) to further illustrate the clinical phenotypic correlation of these factors.

### 3.8. GSEA and HPA

**Figure 8:**
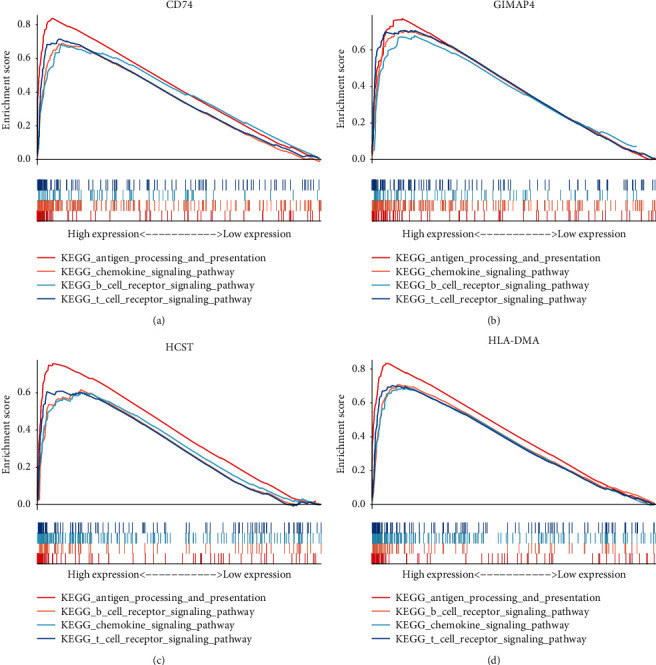
Antigen processing and presentation, the chemokine signaling pathway, B cell receptor signaling pathway, and the T cell receptor signaling pathway were related to the high expression group in CD74, GIMAP4, HCST, and HLA-DMA.

Antigen processing and presentation, the chemokine signaling pathway, B cell receptor signaling pathway, and the T cell receptor signaling pathway were related to the high expression group in CD74, GIMAP4, HCST, and HLA-DMA (Figure 8).

**Figure 9:**
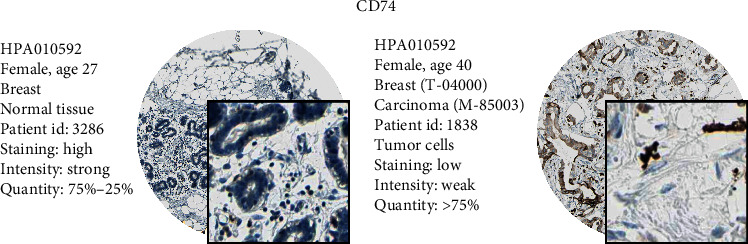
We compared the various expression levels of these genes between normal and tumor tissues. HPA010592 was the antibody of CD74, which showed higher intensity in the tumor tissue against normal tissue.

We compared the various expression levels of these genes between normal and tumor tissues. Labeling with HPA010592, an antibody against CD74, showed higher intensity in the tumor tissue than that in normal tissue (Figure 9).

## 4. Discussion

In 2000, immune checkpoint inhibitors were used to enhance the antitumor ability of the immune system and achieved a better curative effect. In 2011, the world's first immune checkpoint inhibitor, cytotoxic T lymphocyte-associated antigen 4 (CTLA-4) antibody, was approved by the U.S. Food and Drug Administration, marking a new era in tumor immunotherapy. Checkpoint inhibitors and programmed cell death receptor-1 (PD-1) antibodies increase the 5-year survival rate of relapsed/refractory non-small cell lung cancer from 5% to 16% [24]. Tumor immunotherapy is further promoted to the status of a research hotspot, such that immunotherapy has become the main treatment for some tumors. Biomarkers closely related to immunotherapy include PD-L1 IC (IC immune cell), PD-L1 TC (tumor cell), CD8+ T cells, tumor mutational burden, and others. Their expression levels and roles differ in various stages of particular tumors. It has been reported that immunotherapy is more effective in the context of T cell proliferation [25]. For this reason, we intended to identify the most relevant biological processes and coexpression networks in the tumor microenvironment with CD8+ T lymphocyte infiltration and to determine whether these biological processes and related factors would improve outcomes by promoting CD8+ T lymphocyte infiltration.

We obtained 1,097 breast cancer samples through TCGA-BRCA and constructed a CD8+ T-related coexpression network by estimating the proportion of CD8+ T lymphocytes in each sample. WGCNA identifies gene modules with similar expression patterns. By calculating the correlation between multiple modules and CD8 content, the most relevant biological functions and coexpression networks of CD8+ T lymphocytes can be screened out. We determined that the 25 factors in the yellow module were most closely related to the content of CD8+ T lymphocytes and were most related to the antigen presentation process and interferon responses. Studies showed that the drug resistance of PD-1 or PD-L1 monoclonal antibody after immunotherapy was closely related to tumor antigen mutation and antigen presentation process [26]. In addition, the tumor interferon signaling pathway was related to the multigene resistance program that blocks immune checkpoints [27].

Twenty-five factors can be used as independent prognostic protective factors in breast cancer patients. This conclusion is consistent with the trend of the influence of CD8+ T lymphocytes on outcomes. Then, we constructed a lasso regression model consisting of eight factors, based on the 25 independent prognostic factors. CD74, GIMAP4, HCST, and HLA-DMA not only showed good clinical phenotype correlation but also correlated with M2 type macrophages, tumor purity, and immune score.

CD74 encodes a protein related to major histocompatibility complex (MHC) class II that regulates immune response antigen presentation. As the cell surface receptor of the cytokine macrophage migration inhibitory factor, CD74 initiates survival pathways and cell proliferation when combined with the encoded protein [28]. Basha et al. demonstrated the CD74-dependent MHC class I cross-presentation pathway in dendritic cells, which is thought to be related to the response of MHC class I restricted cytolytic T lymphocytes (CTL) to cell-related antigens [29]. GIMAP4, also known as GTPase, encodes proteins belonging to the immune-related nucleotide (IAN) subfamily of GTP-binding superfamily and nucleotide-binding proteins. Mégarbané et al. found that the lack of GIMAPs may play a tumor-suppressive role against breast cancer [30]. The HCST encodes a transmembrane signaling adapter that encodes a protein that forms part of the immune recognition receptor complex with type C lectin-like receptor NKG2D [31]. Gilfillan et al. found that, in dap10-deficient mice, CD8+ T cells lack NKG2D expression and cannot mediate tumor-specific responses [32]. HLA-DMA is also called MHC class II, DM alpha [33]. Both HLA-DMA and HLA-DMB genes are needed for the formation of MHC class II/peptide complexes in antigen-presenting cells [34].

**Figure 10:**
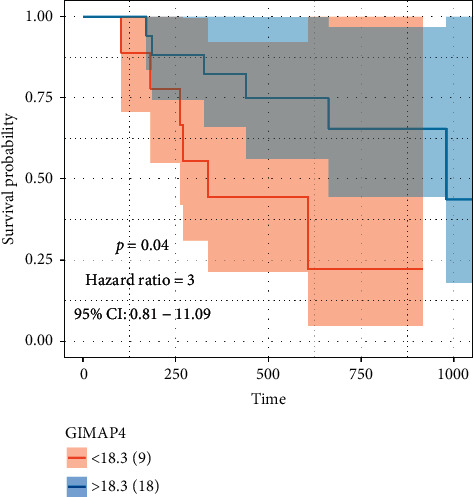
GIMAP4 can be used as an independent prognostic factor after immunotherapy.

To determine whether coexpression of CD8+ T lymphocytes improves the inference of immunotherapy, we attempted to identify the cohort with immunotherapy outcomes. Unfortunately, we did not find such breast cancer results. We only found the factors in a follow-up cohort of immunotherapy for melanoma and found that GIMAP4 can be used as an independent prognostic factor after immunotherapy (Figure 10).

This article has certain limitations. First, only samples from two cohorts were included, and joint analysis is still needed for more cohorts. In addition, this article only explains the coexpression network that promotes the infiltration of CD8+ T lymphocytes from the perspective of computational biology. More in-depth cell labeling experiments need to be performed. In the end, we did not obtain enough immunotherapy follow-up samples, and statistical systematic errors are inevitable.

These eight CD8+ T lymphocyte proportion coexpression genes increase CD8^+^ T lymphocyte in breast cancer by an antigen presentation process. The mechanism might provide new ideas to improve the curative effect in patients who do not benefit from immune therapy.

## Figures and Tables

**Table 1:** The module and gene significance for CD8^+^ T cells related genes.

ID	ModuleColor	GS.TcellsCD8	p.GS.TcellsCD8
CD8A	Yellow	0.637280634	3.56*E* − 77
GZMA	Yellow	0.615288086	1.27*E* − 70
NKG7	Yellow	0.596372475	2.19*E* − 65
CST7	Yellow	0.543848238	1.57*E* − 52
CD2	Yellow	0.523524185	3.85*E* − 48
CD3D	Yellow	0.505128651	2.05*E* − 44
GZMK	Yellow	0.493345337	3.81*E* − 42
GZMB	Yellow	0.489263621	2.22*E* − 41
CD3E	Yellow	0.485176437	1.27*E* − 40
CCL5	Yellow	0.484214853	1.91*E* − 40
CXCL9	Yellow	0.466110209	3.17*E* − 37
IRF1	Yellow	0.456695053	1.26*E* − 35
HLA-E	Yellow	0.449975369	1.64*E* − 34
HLA-DPB1	Yellow	0.447710011	3.83*E* − 34
CD74	Yellow	0.446926288	5.13*E* − 34
IL2RG	Yellow	0.443336997	1.94*E* − 33
PSMB9	Yellow	0.429897672	2.47*E* − 31
CD27	Yellow	0.426640739	7.73*E* − 31
GBP4	Yellow	0.422872734	2.85*E* − 30
CORO1A	Yellow	0.42090121	5.61*E* − 30
PSMB10	Yellow	0.419193325	1.00*E* − 29
GIMAP4	Yellow	0.416341962	2.63*E* − 29
CD48	Yellow	0.416335453	2.64*E* − 29
HLA-DMA	Yellow	0.4146621	4.63*E* − 29
SELPLG	Yellow	0.414330462	5.17*E* − 29
TNFRSF1B	Yellow	0.413004893	8.05*E* − 29
HCST	Yellow	0.406386032	7.12*E* − 28
GMFG	Yellow	0.403523457	1.80*E* − 27

GS : gene significance.

**Table 2:** The best prognosis-related model results selected by Rbsurv package in R.

Order	Gene	nloglik	AIC	Selected
0	0	285.87	752.73	
1	CD74	267.7	715.41	*∗*
2	IRF1	252.02	708.03	*∗*
3	CCL5	250.31	685.62	*∗*
4	GIMAP4	246.07	656.15	*∗*
5	HCST	245.42	623.76	*∗*
6	CST7	242.89	608.11	*∗*
7	PSMB10	242.24	592.05	*∗*
8	HLA-DMA	242.11	565.02	*∗*
9	IL2RG	241.68	541.32	*∗*
10	CD3E	240.76	531.97	*∗*
11	SELPLG	240.65	523.94	*∗*
12	CORO1A	239.77	513.5	
13	CD3D	239.62	508.48	
14	GZMK	239.59	506.19	
15	CD48	236.85	504.99	
16	PSMB9	236.21	495.24	
17	CD2	213.77	493.53	
18	GBP4	203.21	480.42	
19	CD8A	202.82	470.63	

AIC: akaike information criterions; nloglik: negative log-likelihood.

## Data Availability

The datasets TCGA-BRCA for this study can be found in The Cancer Genome Atlas (http://cancergenome.nih.gov/). The datasets GSE78220 in this study can be found in the GEO (http://www.ncbi.nlm.nih.gov/geo/).

## Supplementary Materials

Supplementary Materials Supplementary Table 1: the baseline information of TCGA-BRCA.
