# Supplementary material for: CD8+ T Lymphocyte Coexpression Genes Correlate with Immune Microenvironment and Overall Survival in Breast Cancer
Source: J Oncol. 2021 Mar 27;2021:5533923. doi: 10.1155/2021/5533923 (PMC8019641; doi:10.1155/2021/5533923)
Supplement: Supplementary Materials — Supplementary Table 1: the baseline information of TCGA-BRCA. [file 5533923.f1.docx]

Supplementary Table 1

|  | group | number |
| --- | --- | --- |
| age | >60 | 493 |
|  | <60 | 583 |
| ER status | ER pos | 808 |
|  | ER neg | 240 |
| stage | stage I | 183 |
|  | stage II | 622 |
|  | stage III | 250 |
|  | stage IV | 34 |
| vital status | alive | 945 |
|  | dead | 152 |
| gender | male | 12 |
|  | female | 1085 |
| cancer_status | tumor free | 935 |
|  | with tumor | 126 |
